# Fatigue and Human Performance: An Updated Framework

**DOI:** 10.1007/s40279-022-01748-2

**Published:** 2022-10-18

**Authors:** Martin Behrens, Martin Gube, Helmi Chaabene, Olaf Prieske, Alexandre Zenon, Kim-Charline Broscheid, Lutz Schega, Florian Husmann, Matthias Weippert

**Affiliations:** 1 Department of Sport Science, Institute III, Otto-Von-Guericke University Magdeburg, Zschokkestraße 32, 39104 Magdeburg, Germany; 2 Department of Orthopedics, Rostock University Medical Center, Rostock, Germany; 3 Department of Sport Science, University of Rostock, Rostock, Germany; 4 Department of Sports and Health Sciences, Faculty of Human Sciences, University of Potsdam, Potsdam, Germany; 5 Division of Exercise and Movement, University of Applied Sciences for Sports and Management Potsdam, Potsdam, Germany; 6 Institut de Neurosciences Cognitives et Intégratives d’Aquitaine (INCIA)-UMR 5287, CNRS, University of Bordeaux, Bordeaux, France; 7 Institute for Applied Training Science, Leipzig, Germany

## Abstract

Fatigue has been defined differently in the literature depending on the field of research. The inconsistent use of the term fatigue complicated scientific communication, thereby limiting progress towards a more in-depth understanding of the phenomenon. Therefore, Enoka and Duchateau (Med Sci Sports Exerc 48:2228–38, 2016, [3]) proposed a fatigue framework that distinguishes between trait fatigue (i.e., fatigue experienced by an individual over a longer period of time) and motor or cognitive task-induced state fatigue (i.e., self-reported disabling symptom derived from the two interdependent attributes performance fatigability and perceived fatigability). Thereby, performance fatigability describes a decrease in an objective performance measure, while perceived fatigability refers to the sensations that regulate the integrity of the performer. Although this framework served as a good starting point to unravel the psychophysiology of fatigue, several important aspects were not included and the interdependence of the mechanisms driving performance fatigability and perceived fatigability were not comprehensively discussed. Therefore, the present narrative review aimed to (1) update the fatigue framework suggested by Enoka and Duchateau (Med Sci Sports Exerc 48:2228–38, 2016, [3]) pertaining the taxonomy (i.e., cognitive performance fatigue and perceived cognitive fatigue were added) and important determinants that were not considered previously (e.g., effort perception, affective valence, self-regulation), (2) discuss the mechanisms underlying performance fatigue and perceived fatigue in response to motor and cognitive tasks as well as their interdependence, and (3) provide recommendations for future research on these interactions. We propose to define motor or cognitive task-induced state fatigue as a psychophysiological condition characterized by a decrease in motor or cognitive performance (i.e., motor or cognitive performance fatigue, respectively) and/or an increased perception of fatigue (i.e., perceived motor or cognitive fatigue). These dimensions are interdependent, hinge on different determinants, and depend on body homeostasis (e.g., wakefulness, core temperature) as well as several modulating factors (e.g., age, sex, diseases, characteristics of the motor or cognitive task). Consequently, there is no single factor primarily determining performance fatigue and perceived fatigue in response to motor or cognitive tasks. Instead, the relative weight of each determinant and their interaction are modulated by several factors.

## Key Points

**Table:** 

Motor or cognitive task-induced state fatigue can be defined as a psychophysiological condition characterized by a decrease in motor or cognitive performance (i.e., motor or cognitive performance fatigue, respectively) and/or an increased perception of fatigue (i.e., perceived motor or cognitive fatigue).
Performance fatigue and perceived fatigue are interdependent, hinge on different determinants, and depend on several modulating factors (e.g., age, sex, diseases, characteristics of the motor or cognitive task).
The combined monitoring of performance fatigue and perceived fatigue measures as well as the investigation of the underlying mechanisms will help to unravel the interactions between the different dimensions of fatigue and their impact on human performance. This will contribute to assess the relative weight of each determinant and their interactions depending on several modulating factors.

## Introduction

The capacity to maintain intense and/or sustained motor and cognitive tasks is important in human life and is required during daily, physical, vocational, and educational activities. The multitude of psychophysiological processes that inevitably accompany motor or cognitive activity above a certain intensity or duration can become a limiting factor for motor as well as cognitive performance and are typically summarized under the umbrella term fatigue. In the past, a variety of disciplines (e.g., psychology, exercise physiology, neuroscience, medical fields) have specialized on selected aspects investigating either the subjective perception of fatigue or changes in motor or cognitive performance [1–5]. Due to this fragmentation, a multitude of fatigue definitions emerged leading to an inconsistent use of the term and neglecting the dynamic interactions between the task-induced psychophysiological adjustments and the resulting perceptual, affective, and cognitive responses. Therefore, mechanistic insights into the psychophysiological processes associated with fatigue in healthy and clinical populations as well as the development of effective interventions were hampered [2, 3]. This is not only crucial to increase the performance of athletes and healthy people, but it is also important for vulnerable, deconditioned, as well as clinical populations due to fatigue-induced negative effects on the motor and cognitive capacity as well as quality of life.

To resolve the ambiguity of fatigue definitions, Enoka and Duchateau [3] proposed a framework defining fatigue as a self-reported disabling symptom that limits physical and cognitive functions due to interactions between performance fatigability (i.e., decrease in an objective performance measure) and perceived fatigability (i.e., changes in the sensations that regulate the integrity of the performer). Both performance fatigability and perceived fatigability depend on several factors that determine the decline in motor performance (i.e., muscle activation and contractile function) as well as the changes in the individuals’s sensations (i.e., the psychological and homeostatic state of the individual). In their framework, the authors highlighted the interdependence of performance fatigability and perceived fatigability with both contributing to the self-reported symptom fatigue. The advantage of this framework is its applicability to both healthy and clinical populations, since it refers to the fatigue mechanisms whose relative weight is subject- and task-dependent.

Although the fatigue framework suggested by Enoka and Duchateau [3] served as a good starting point to unravel the psychophysiology of fatigue induced by motor and cognitive tasks, several important aspects were not included and discussed (e.g., effort perception, affective valence, self-regulation). Moreover, the authors’ definition of fatigue comprised also a decline in cognitive performance, which was not adequately embedded in their framework in terms of the taxonomy and the underlying mechanisms. Finally, the interdependence of the mechanisms driving performance fatigability and perceived fatigability as well as the need to thoroughly quantify these aspects were not comprehensively discussed.

Therefore, the present narrative review aimed at (1) updating the framework and definition of fatigue proposed by Enoka and Duchateau [3] pertaining the taxonomy (i.e., cognitive performance fatigue and perceived cognitive fatigue were added) and important determinants that were not considered previously (i.e., effort perception, affective valence, self-regulation, and time perception), (2) discussing the mechanisms driving performance fatigue and perceived fatigue in response to motor and cognitive tasks as well as their interdependence, and (3) providing recommendations for future research on these interactions.

## Taxonomy of Fatigue

To precisely define fatigue, it is first important to differentiate between trait fatigue and state fatigue. Trait fatigue describes the fatigue experienced by an individual over a longer period of time (e.g., weeks and months), which is relatively stable. Trait fatigue is a symptom associated with many diseases (e.g., multiple sclerosis, chronic obstructive pulmonary disease, rheumatoid arthritis) and is a result of primary disease-related mechanisms (e.g., neurodegeneration, inflammation) as well as secondary mechanisms not directly caused by the disease but associated with it (e.g., depression, sleep problems, medication) [2, 6–8]. However, trait fatigue can also be present in a milder form in healthy people [9].

**Fig. 1:**
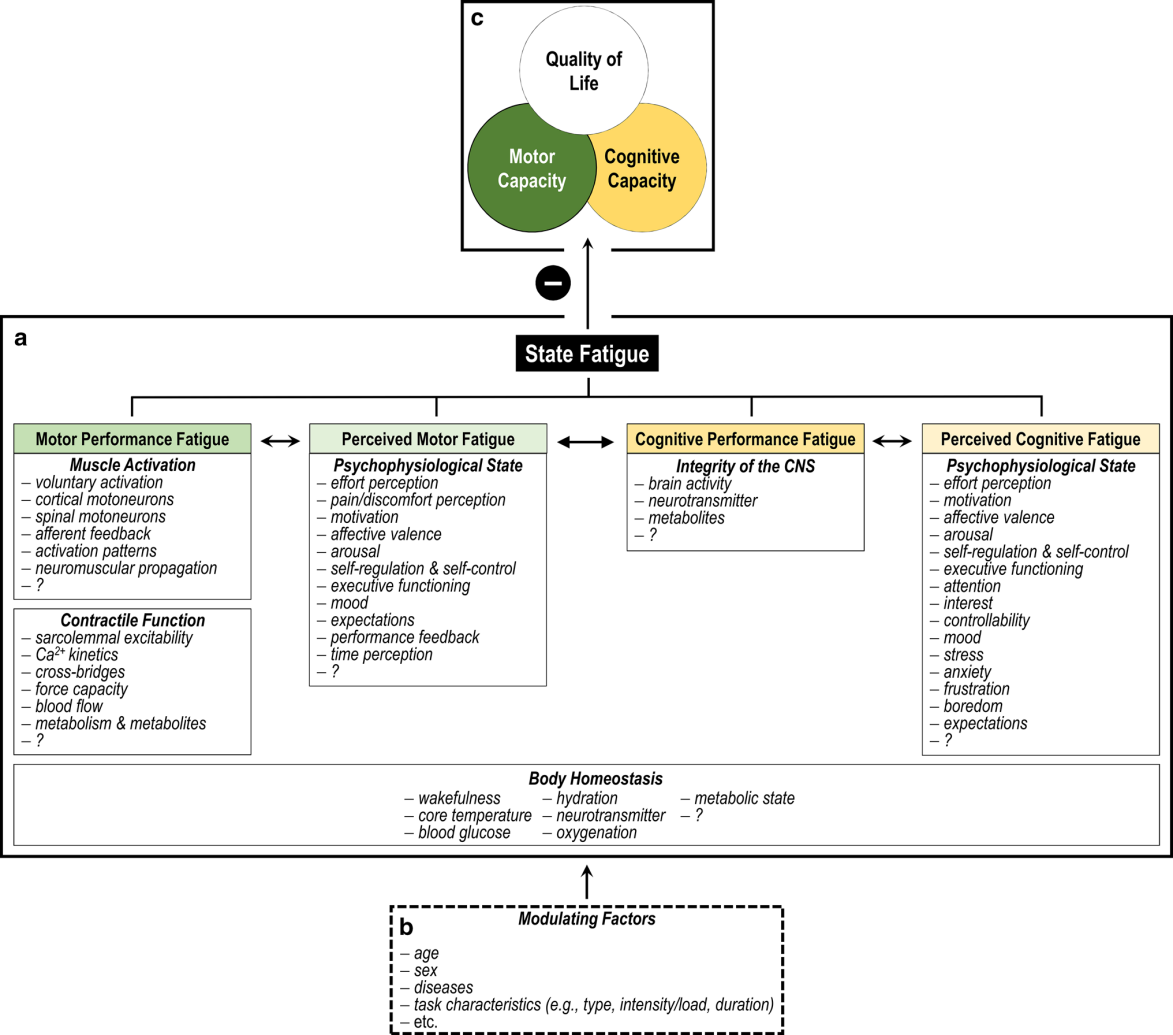
Adapted motor and/or cognitive task-induced state fatigue framework with its interdependent dimensions and the respective determinants first proposed by Enoka and Duchateau [3] (**a**). The extent of state fatigue mirrored by these dimensions depends on several modulating factors (**b**) and can have negative consequences for the motor and cognitive capacity, which might negatively affect quality of life (**c**) particularly in vulnerable, deconditioned, and clinical populations. The bidirectional arrows indicate the interdependence between all dimensions. Please note that effort perception, affective valence, self-regulation and self-control, as well as time perception were added to the potential determinants of perceived motor fatigue compared to the framework of Enoka and Duchateau [3]. Furthermore, cognitive performance fatigue, perceived cognitive fatigue, and the potentially contributing factors were added to the framework. *CNS* central nervous system, *?* unknown factors that should be added in the future

Activity-induced state fatigue, in turn, is characterized by an acute and temporary change in motor or cognitive performance as well as the subjective experience of weariness or exhaustion that occur in the context of a specific motor or cognitive task [3, 7, 10–12]. Inspired by the definition of fatigue proposed by Enoka and Duchateau [3], we suggest to define motor and cognitive task-induced state fatigue as a psychophysiological condition that is characterized by a decrease in motor or cognitive performance and/or an increased perception of fatigue. The acute reduction in motor and cognitive performance can be labeled as motor and cognitive performance fatigue, respectively. While motor performance fatigue (e.g., decrease in maximal voluntary force) depends on factors contributing to muscle activation and contractile function, the precise origin of cognitive performance fatigue (e.g., decrease in reaction time) remains disputed, but may depend on the integrity of the central nervous system. The motor and cognitive task-induced modulation of the perception of fatigue can be termed perceived motor and cognitive fatigue, respectively, which depend on the psychophysiological state of the individual. Motor and cognitive performance fatigue as well as perceived motor and cognitive fatigue further depend on factors related to body homeostasis, are interdependent, and hinge on different determinants (Fig. 1a). Thereby, the extent of motor and cognitive performance fatigue as well as perceived motor and cognitive fatigue depends on several modulating factors (e.g., characteristics of the subject and the task) (Fig. 1b) and can have detrimental effects on the motor and cognitive capacity of humans. In the long term, this can result in a reduced quality of life, particularly in vulnerable, deconditioned, and clinical populations (Fig. 1c) [3, 7].

Of note, this definition slightly differs from the taxonomy provided by Enoka and Duchateau [3], who have defined state fatigue as a self-reported disabling symptom derived from the interdependent attributes performance fatigability and perceived fatigability. However, this definition introduces the problem that state fatigue is assessed by self-report, which is also reflected by quantifying perceived fatigability (i.e., the task-induced rate of change in perceived fatigue [13]). In addition, performance may decrease without a corresponding increase in the perception of fatigue or vice versa. This potential selective change is not captured by the definition of Enoka and Duchateau [3]. In addition, since we do not refer to state fatigue as a self-reported disabling symptom, the term fatigability does not seem to be necessary as it does not contribute any benefit compared to the term fatigue. In fact, the term fatigue was also formerly used to describe a decrease in performance (e.g., muscle fatigue, cognitive fatigue), physiological function (e.g., central fatigue, peripheral fatigue), or an increase in the perception of fatigue (e.g., mental fatigue) [1, 10, 14–18]. The terms fatigability and fatigable could, however, be used synonymously as linguistic variations (e.g., when subjects have a high-performance fatigability or persons are highly fatigable). Of note, in the following paragraphs, the proposed fatigue taxonomy was applied, even when the cited studies have not used this terminology.

The psychophysiological alterations during fatiguing motor exercise can be interpreted as a protective mechanism that regulate exercise behavior to ensure the preservation of homeostasis of various physiological systems in the human body [15, 19, 20].

This is in contrast to fatigue resulting from sustained cognitive tasks, the psychophysiological underpinnings of which remain unclear. While some have proposed that the ultimate function of cognitive fatigue would be to redirect behavior from the current to more rewarding and/or less effortful activities [21, 22], others have argued, in closer agreement with motor fatigue, that it is a protective mechanism urging people to stop the present activity in anticipation of future adverse, functional consequences [23, 24].

## Motor Performance Fatigue and Perceived Motor Fatigue

### Motor Performance Fatigue

Motor performance fatigue (traditionally termed muscle or neuromuscular fatigue) can be quantified as a decrease in maximal voluntary force production capacity of the neuromuscular system, which is determined by neural and muscular factors [3]. Depending on the characteristics of the motor task (e.g., duration, intensity) and other factors (e.g., age, sex, diseases, fitness level), the underlying mechanisms of motor performance fatigue include changes at distinct levels within the neuromuscular system that are involved in muscular force production and thus movements. These include, but are not limited to: (1) excitability of the motor cortex, (2) descending corticospinal transmission of action potentials, (3) excitability of spinal α-motoneurons, (4) neuromuscular transmission, (5) sarcolemmal excitability, (6) propagation of action potentials into the transverse tubular system, (7) intracellular calcium ion (Ca^2+^) kinetics, and (8) force production within the cross-bridge cycle [14, 25, 26]. Intense and/or sustained motor tasks can impair these physiological processes, which in turn, can contribute to a reduced motor performance. To determine the origin of these changes within the neuromuscular system, a distinction between neural (central) and muscular (peripheral) determinants of motor performance fatigue has been established. Neural determinants include aspects related to muscle activation (traditionally termed central fatigue) that can change during a motor task (Fig. 1a). These changes comprise decrements in voluntary activation of individual muscles associated with modulations in cortical motoneurons and/or spinal α-motoneurons. The exercise-induced neural as well as muscular alterations lead to task-specific adaptations in the firing frequency and/or recruitment of motor units. In this context, various processes play a role including the modulation of intrinsic properties of motoneurons, an increase in inhibitory afferent feedback from group III and IV muscle afferents, a decrease in facilitatory afferent feedback, and changes in neuromodulators [16]. In addition, activation patterns of synergistic and antagonistic muscles can change during a fatiguing motor task, which in turn can negatively affect intermuscular coordination and thus force production capacity [27, 28].

Beside these neural determinants, changes in the contractile function of muscles can contribute to the extent of motor performance fatigue (Fig. 1a). The impairment of contractile function largely depends on muscle perfusion and the intramuscular metabolism. For instance, intense motor tasks lead to an increased accumulation of metabolites (e.g., inorganic phosphate, reactive oxygen species, hydrogen ions) that can impair contractile function of muscles. Under physiological conditions, inorganic phosphate seems to be primarily responsible for the reduction in contractile function, while reactive oxygen species seems to be involved in the prolonged force depression after exercise [25, 26, 29, 30]. The main factors that determine the decrease in contractile function and thus the contractile force of muscles are reductions in sarcolemmal excitability, Ca^2+^ release from the sarcoplasmic reticulum, myofibrillar Ca^2+^ sensitivity, and the force-generating capacity of the cross-bridges per se [3, 14, 25, 26, 31]. Importantly, the decrease in muscle activation and/or contractile function of muscles during and after exercise is sensitive to different homeostatic perturbations like hyperthermia [32], hypoxia [33], and hypoglycemia [34].

### Perceived Motor Fatigue

Perceived motor fatigue refers to the increase in the subjective perception of fatigue emerging during a motor task that can affect motor task performance [5, 13]. It is often defined as a transient sensation of tiredness, weariness, lack of energy, or exhaustion [35, 36]. Recently, it was proposed to define perceived fatigue as the feeling of a need to rest or a mismatch between effort expended and actual performance [36].

**Fig. 2:**
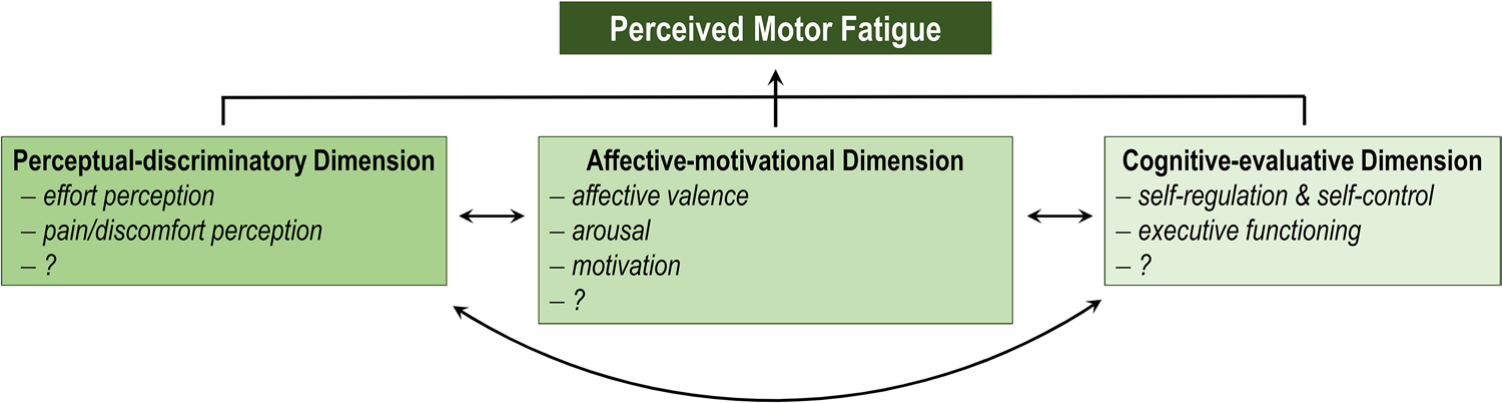
Adapted three-dimensional dynamical system framework of perceived motor fatigue first proposed by Venhorst et al. [5]. The bidirectional arrows indicate the interdependence between the dimensions

Irrespective of the specific definition, the nature and extent of perceived motor fatigue depend on the psychophysiological state of the individual, which shapes the perceptual, affective, and cognitive processes during exercise (Fig. 1a). For example, exercising above an individual critical threshold (e.g., above critical power [37]) leads to metabolite accumulation resulting in a decline in contractile function of muscles [26]. Therefore, an increased muscle activation signal is necessary to maintain the submaximal force output, which is associated with an increased effort perception [38]. Besides this, exercise-induced pain and discomfort arise as a result of the enhanced metabolite accumulation, breathing rate, and body temperature [39]. These perceptual responses to exercise make a person feel increasingly bad and require regulatory cognitive processes to avoid slowing down or stopping the motor task [5, 40, 41]. Recently, Venhorst et al. [5] proposed a three-dimensional dynamical system framework to better understand these psychophysiological determinants of perceived motor fatigue. It allows the classification of some of the determining factors of perceived motor fatigue into three dimensions. Following this framework, the perceptual responses to exercise (e.g., effort perception, exercise-induced pain/discomfort perception) can be attributed to (1) the perceptual-discriminatory dimension. The intensity of these perceptions has an impact on (2) the affective-motivational dimension (e.g., affective valence, arousal, motivation). The motor task-induced changes in these dimensions strongly determine the processes in (3) the cognitive-evaluative dimension related to the decision to slow down or speed up (pacing behavior) or even to disengage from exercise. These processes involve, for instance, self-regulation, self-control, and executive functioning (Fig. 2). This three-dimensional dynamical system framework allows the comprehensive as well as specific assessment of the factors determining perceived motor fatigue and contributes to the understanding of the strain-perception-thinking-action coupling during fatiguing exercise [5]. However, the interactions between the perceptual-discriminatory dimension, the affective-motivational dimension, and the cognitive-evaluative dimension should not be regarded as hierarchical but as interdependent.

#### Perceptual-Discriminatory Dimension of Perceived Motor Fatigue

The effort perceived during a motor task can be attributed to the perceptual-discriminatory dimension and is associated with perceived motor fatigue [16, 42]. Moreover, motor task-related effort perception is considered an important determinant of exercise behavior and endurance performance [5, 43, 44]. Of note, there is controversy about whether effort perception results from centrally mediated feedforward mechanisms (i.e., corollary discharge model) and/or afferent feedback from the working and respiratory muscles (i.e., afferent feedback or combined model). However, it is well accepted that processing of sensory signals in the brain is involved [38, 45]. Effort perception, along with motivation, is one core element of the psychobiological model of endurance performance [43, 46] and it has been shown that interventions, which reduced effort perception during a sustained motor task, have subsequently led to an increased exercise tolerance (e.g., time to exhaustion during submaximal exercise) [47–52]. Conversely, effort perception during endurance exercise was higher and motor performance was reduced after interventions inducing homeostatic perturbations like hypoglycemia [34], hyperthermia [53], dehydration [54], hypoxia [55], and sleep deprivation [56].

A less studied factor that also belongs to the perceptual-discriminatory dimension and can influence perceived motor fatigue is exercise-induced muscle pain/discomfort. For instance, intense motor tasks lead to the accumulation of metabolites in the extracellular environment, resulting in an increased exercise-induced muscle pain perception, due to the activation of group III and IV muscle afferents [39]. Acute interventions aiming to reduce exercise-induced muscle pain have been shown to improve performance during sustained submaximal motor tasks [57], whereas artificially increasing exercise-induced muscle pain had the opposite effect [58]. These examples provide evidence for the importance of exercise-induced perceptual responses (e.g., effort and exercise-induced pain) for motor task performance.

#### Affective-Motivational Dimension of Perceived Motor Fatigue

The intensity of the perceptual responses (e.g., effort and exercise-induced pain/discomfort) has effects on the affective state and the motivation of an individual, which can be attributed to the affective-motivational dimension. The affective state of an individual also contributes to perceived motor fatigue and can influence exercise behavior as well as time to exhaustion during motor tasks [5, 42, 59]. It is thought that ratings of affective valence and arousal can mirror the affective state of individuals. Affective valence reflects how a person currently feels in general (i.e., from very good to very bad) [60]. These states are thought to be subjective indicators of the homeostatic status during motor tasks mediated by afferent nerve fibers that detect the mechanical, thermal, chemical, metabolic, and hormonal state of various tissues. Their projections to different brain areas (e.g., anterior insula, anterior cingulate cortex) enable conscious awareness of these stimuli and may serve as a protective mechanism for the body [61, 62]. Consequently, during fatiguing motor tasks, exercise intensity-dependent homeostatic perturbations in the respective physiological subsystems can contribute to the development of an acute negative affective valence. This is the case, for example, during the transition from the relative dominance of the aerobic to anaerobic muscle metabolism during exercise [40, 63]. Furthermore, other homeostatic perturbations, such as glycogen depletion, can also accelerate the development of negative affective valence during submaximal constant-load endurance exercise and shorten the time to exhaustion during this motor task [59]. Interestingly, the authors have found that the rate of decline in affective valence was highly and positively correlated with time to exhaustion. These findings again highlight the relevance of aspects of perceived motor fatigue for motor task performance.

#### Cognitive-Evaluative Dimension of Perceived Motor Fatigue

The changes in the perceptual-discriminatory and the affective-motivational dimensions influence processes within the cognitive-evaluative dimension with consequences for task performance during fatiguing exercise [5]. In this regard, the role of the self-regulation capacity of an individual for endurance performance has been discussed [41]. Self-regulation describes the process of bringing thinking and behavior in line with the desired goal. In certain circumstances, it requires self-control [64], which describes the process of overriding one’s predominant (pre-potent, automatic) response tendencies in service of an overarching goal [65]. With regard to motor tasks, individuals have to continuously self-regulate different affective states induced by different perceptions (e.g., effort perception, exercise-induced pain perception), thoughts (e.g., related to task-termination or distractors), and behaviors (e.g., stopping the task or increasing the effort), with consequences for their motor performance. Self-regulation is effortful and relies on the integrity of executive functioning and in particular on the core executive functions, which can be classified into inhibitory control (i.e., response inhibition and interference control), working memory, and cognitive flexibility [66]. During endurance exercise, for instance, the performer has to block numerous distractors to achieve the goal attainment strategy retained in the working memory. Moreover, the individual has to resist the temptation to slow down when he/she is fatigued (inhibition) and might adjust his/her strategy for goal pursuit (cognitive flexibility) [41]. Recent findings support this view and have found that anodal transcranial direct current stimulation (tDCS) applied to the left dorsolateral prefrontal cortex improved Stroop task performance, a measure of inhibitory control, and time to exhaustion during submaximal constant-load cycling. Further, effort perception was lower, which was ascribed to the tDCS-induced increase in neural excitability of the target areas [67]. Of note, there are also findings that tDCS did not modulate motor performance and exercise-related sensations [68].

In addition to the mentioned key-determinants of perceived motor fatigue, other important aspects contribute to the psychophysiological state of an individual and have an impact on perceived motor fatigue. These include mood, expectations, the presence of performance feedback, and time perception [3, 39, 69]. Besides, there are further factors contributing to perceived motor fatigue, which should be added to the list of determinants of perceived motor fatigue in the future, taking the three-dimensional dynamical system framework of perceived motor fatigue into account (Fig. 2).

### Modulating Factors of Motor Performance Fatigue and Perceived Motor Fatigue

There are various modulating factors that can influence the extent of motor performance fatigue and perceived motor fatigue (Fig. 1b). The main subject-specific factors include age, sex, the presence of diseases, and the physical fitness level. The extent of fatigue in the different domains is further determined by the characteristics of the motor task (e.g., duration, intensity, contraction mode, and velocity), environmental conditions (e.g., temperature, oxygen availability), the context (e.g., competition), as well as acute and chronic interventions (e.g., ergogenic supplements, training interventions) [2, 3, 7, 16, 31, 51, 70–73]. In this article, we will only discuss the most studied subject- and motor task-specific factors that can modulate motor performance fatigue and perceived motor fatigue.

#### Subject-Specific Factors and Motor Performance Fatigue

In terms of subject-specific factors, it is well-known that motor function declines when people get older due to structural and functional changes within the neuromuscular system (e.g., decline in muscle quantity, quality, and function as well as neural drive) [74–76]. Interestingly, older adults often exhibit less motor performance fatigue during submaximal isometric contractions compared to young adults. This might be due to slower contractile properties requiring lower motor unit firing frequencies to reach a tetanic force output. Moreover, older adults possess a lower percentage of type II muscle fibers and a reduced reliance on glycolytic metabolism that preserves the contractile function of muscles during this type of exercise [71]. However, motor performance fatigue after fast concentric contractions is higher in older compared to younger adults [77]. This can most likely be attributed to the slower shortening velocity of muscle fibers, the loss of high-threshold motor units, less optimal muscle activation during rapid muscle actions, and impairment in skeletal muscle bioenergetics. Nevertheless, these age-related differences strongly depend on the muscle group under investigation [31, 71, 77, 78].

Furthermore, the extent of motor performance fatigue can differ between females and males during fatiguing isometric and dynamic tasks. Males usually show larger motor performance fatigue during single-joint isometric and slow-to-moderate velocity muscle actions as well as whole-body exercise compared to females [31, 79, 80]. It is thought that sex-related differences within the neuromuscular system are responsible for the lower motor performance fatigue of females. For instance, females possess a larger percentage of type I muscle fibers resulting in a higher capillarization and mitochondrial respiratory capacity, a lower glycogen utilization, as well as an increased muscle perfusion compared to males. These physiological differences lead to a slower accumulation of metabolites, and, in turn, a slower decline in contractile force and voluntary activation of muscles. However, the sex difference in motor performance fatigue is diminished when performing fast-velocity muscle actions and strongly depends on the investigated muscle(s) [31, 70, 81].

Motor performance fatigue after physical activity is often more pronounced in clinical populations compared with healthy controls leading to a reduced exercise tolerance and quality of life [2, 82]. The relative contribution of neural (i.e., muscle activation) and muscular factors (i.e., contractile function) to the larger motor performance decline strongly depends on the pathophysiology of the disease. For example, neurologic diseases like multiple sclerosis seem to be associated with a larger motor task-induced decrease in muscle activation characteristics [83, 84], while diseases affecting vascular and muscle functions can impair the contractile function of muscles to a larger extent compared to healthy controls [7, 8, 85]. Therefore, the relative contribution of neural (i.e., muscle activation) and muscular factors (i.e., contractile function) to motor performance fatigue in patients seems to depend on the disease-specific locus of the impairments in the body (primary mechanisms). However, secondary mechanisms related to these impairments, such as reduced physical activity, can also contribute to motor performance fatigue in these populations.

#### Characteristics of the Motor Task and Motor Performance Fatigue

One of the most important and extensively studied factors influencing the extent of motor performance fatigue are the characteristics of the motor task, which determine the stress imposed on the involved physiological subsystems. In this context, the magnitude of the decline in maximal voluntary force and the relative contribution of changes in muscle activation and contractile function strongly depend on the duration and intensity of exercise, the mode and velocity of muscle action, and the involved muscle mass [16, 31, 86–90].

A general finding is that high-intensity exercise of short duration decreases maximal voluntary force primarily due to an impaired contractile function of muscles together with a small reduction in voluntary activation. In contrast, low-intensity exercise tends to provoke a substantial decrease in muscle activation and a smaller reduction in contractile function of the involved muscles. This is partly due to muscle metabolic factors that differ between high- and low-intensity exercise [16, 17, 89]. Further, the contraction mode of a muscle during a motor task can modulate motor performance fatigue and the relative contribution of neural (i.e., muscle activation) and muscular factors (i.e., contractile function) to the impairment in motor performance. For example, it has been shown that concentric contractions induce a greater initial reduction in contractile function than eccentric muscle actions. This was attributed to the higher metabolite accumulation during concentric contractions [86, 91]. In contrast, eccentric muscle actions are often associated with microscopic muscle damage impairing contractile force production [92, 93] and voluntary activation [94] over a longer period of time. Another important factor is the contraction velocity during exercise. It has been shown that fast concentric contractions induce a greater reduction in muscle contractile function but a smaller decrease in muscle activation compared with slow concentric muscle actions, probably due to different metabolic requirements [87]. Additionally, the amount of active muscle mass is of relevance. There is evidence that a higher amount of active muscle mass results in a lower drop in contractile function and larger impairments in voluntary activation. It was speculated that this is partly due to an increased inhibitory feedback from group III and IV muscle afferents associated with the larger active muscle mass [88, 95].

#### Subject-Specific Factors and Perceived Motor Fatigue

In line with motor performance fatigue, subject-specific factors like age, sex, and the presence of diseases might also modulate perceived motor fatigue and its determinants. While there are few data for perceived motor fatigue (i.e., rate of change in perceived fatigue assessed before and after or during motor tasks), the results of studies investigating age-related differences seem inconclusive for effort- and exercise-induced pain/discomfort perception [96–98]. For instance, it was shown that exercise-induced muscle pain during a graded arm crank ergometer exercise test was lower in older than in younger females [97]. In contrast, discomfort associated with breathing (i.e., exertional breathlessness) was higher in older than in younger adults of both sexes during a graded treadmill exercise test [98]. The same inconclusive results exist for sex differences in exercise-related perceptions. While effort perception seems to be similar for males and females during fatiguing exercise [99, 100], exercise-induced pain perception was lower in females during a graded cycle ergometer test [101]. However, discomfort associated with breathing (i.e., exertional breathlessness) was higher in older females than males during graded treadmill running [98]. Therefore, the age- and sex-related differences in perceptual responses to fatiguing motor exercise and their contribution to perceived motor fatigue might strongly depend on the origin of the sensory signal as well as the characteristics of the motor task.

Further, clinical populations might also have an increased motor task-induced perceived fatigue depending on the severity of the disease and the level of disability. For example, it was found that perceived motor fatigue (assessed with a visual analog scale) was higher in individuals with multiple sclerosis compared to healthy controls after low-intensity exercise of the non-dominant hand [102]. Moreover, it was shown that persons with multiple sclerosis had a higher effort perception during intermittent submaximal fatiguing exercise of the first dorsal interosseous muscle [103]. Similar results were found for effort perception during exercise in other patient populations, such as coronary heart disease [104] and females with type 2 diabetes [105]. Further, exercise-induced pain can be exacerbated in some diseases like fibromyalgia [106]. Overall, the larger perceptual responses during exercise might contribute to the increased perceived motor fatigue as well as motor performance fatigue observed in many clinical populations.

#### Characteristics of the Motor Task and Perceived Motor Fatigue

The characteristics of the motor task can also modulate the subjective feeling of fatigue and associated perceptions (e.g., effort, pain/discomfort) during motor task execution. It has been shown that perceived effort and exercise-induced pain were higher during fatiguing concentric compared to eccentric resistance exercise [107]. Furthermore, the amount of active muscle mass involved in a motor task can modulate the perceptual responses to fatiguing exercise. For instance, it was shown that single-leg incremental cycling was associated with a higher perceived effort and exercise-induced pain but lower discomfort associated with breathing (dyspnea) compared to double-leg incremental cycling [108]. The differences in the perceptual responses to exercise depending on the mode of muscle action and the involved muscle mass were always accompanied by different physiological adjustments [107, 108]. Furthermore, the motor task-specific homeostatic regulatory processes related to exercise intensity play an important role for the determinants of perceived fatigue (e.g., effort perception, exercise-induced pain/discomfort, affective valence) [5].

The modulation of the regulatory processes within the involved subsystems (e.g., central nervous system and muscle), for instance, by supplements such as caffeine or dietary nitrate, has been shown to have a positive effect on the various perceptions during exercise (e.g., effort and exercise-induced pain) [51, 109]. In addition, the presence of external stimuli (e.g., verbal motivation, monetary incentives, feedback on performance, auditory and visual stimuli) as well as internal stimuli (e.g., self-talk, intermediate goal setting, visualization strategies) may influence the interpretation of sensory signals and thus the extent of perceived motor fatigue [48, 49, 110].

## Cognitive Performance Fatigue and Perceived Cognitive Fatigue

### Cognitive Performance Fatigue

Cognitive performance fatigue (traditionally termed objective cognitive fatigue) induced by sustained and/or intense cognitive tasks can be quantified as a decline in an objective cognitive performance measure during as well as after a cognitive task (e.g., change in reaction time, its variability, and/or accuracy) [4, 10, 111, 112]. The occurrence and extent of cognitive performance fatigue seem to depend on various modulating factors, for instance, subjects-specific factors (e.g., age, sex, diseases) and the characteristics of the cognitive task (e.g., type of task, duration, cognitive load) [113–118] (Fig. 1). Of note, performing prolonged cognitive tasks does not necessarily result in observable decrements in cognitive performance, which was often attributed to a learning effect or an increased compensatory cognitive effort [119–122].

The psychophysiological processes associated with cognitive performance fatigue are still under debate and include, but are not limited to, an altered brain activation, a loss of motivation, and the deterioration of cognitive resources (e.g., attention) [9, 21, 24, 119, 123]. It has been shown, for instance, that the activity of the dorso lateral prefrontal cortex, anterior cingulate cortex, and the insula can change during the execution of a sustained cognitive task [9, 21, 124]. One influential interpretation of these changes is that, with prolonged execution of a task, the invested effort becomes proportionally larger than the associated benefit/reward [23] and the motivation to engage in the task decreases, resulting in a reduction in performance [9, 21]. Furthermore, it has been argued that performing a prolonged cognitive task competes with the desire for control of action and therefore with other cognitive goals as well as basic emotional and biological needs (e.g., resting or doing nothing). Especially the latter are usually more potent in getting attention compared with cognitive goals, indicating their precedence for motivational processes [119]. This view is supported by experiments that have found a decrease in cognitive task performance with time-on-task, which was reversed by increasing motivation with task rewards [116]. However, this effect is not ubiquitous [125] and when comparing performance before and after fatigue induction under similar motivational conditions, there was no evidence for a recovery of task performance after providing rewards, indicating that motivational changes are not the only cause of cognitive performance fatigue [24, 126]. It has been proposed that the neural mechanisms involved in cognitive performance fatigue include changes in neural activity, neurotransmitters, and metabolites (Fig. 1a) [21, 116, 124, 127–131]. Nevertheless, it has been difficult so far to determine the significance of the observed changes in brain activation in relation to the development of cognitive performance fatigue, since they may reflect (1) a deteriorated function of the neural systems required for cognitive task performance, (2) the involvement of brain structures in the monitoring of effort and fatigue, (3) other time-dependent processes like learning, and/or (4) compensatory engagement of brain areas to maintain performance [132]. Of note, alterations in body homeostasis of individuals can modulate the neurophysiological adjustments and thus the cognitive performance changes during fatiguing cognitive tasks as shown, for instance, after inducing hyperthermia [133] and sleep deprivation [134] or after mouth rinsing with caffeine-maltodextrin [135].

### Perceived Cognitive Fatigue

Perceived cognitive fatigue (traditionally termed subjective cognitive or mental fatigue) refers to the increase in the subjective perception of fatigue that develops during the execution of sustained and/or intense cognitive tasks [9, 10, 21]. It is often characterized as feelings of tiredness, weakness, or even exhaustion as well as an aversion to continue with the present task [9, 21]. Recently, it was proposed to define perceived cognitive fatigue as the feeling of a need to rest or a mismatch between effort expended and actual performance [36]. Regardless of the specific definition, the extent of perceived cognitive fatigue depends on the psychophysiological state of the individual that can change throughout a cognitive task and the body homeostasis (Fig. 1a). Similar to cognitive performance fatigue, it was proposed that perceived cognitive fatigue may arise as the consequence of the analysis of the costs and benefits of expending energy in a certain cognitive task [136], and would depend on modification in neurotransmitter release [21]. Performing a difficult sustained cognitive task requires cognitive effort, which would be perceived as increasingly aversive over time [22, 137] and would outweigh the potential task benefits/rewards at a certain point in time (e.g., short- or long-term rewards, negative consequences if the task is terminated, or when the task is intrinsically motivating). Perceived cognitive fatigue would thus serve as a mechanism that would stop or change ongoing behavior when no longer beneficial. Of note, an alternative view on the origin and role of perceived cognitive fatigue is that of an anticipatory protection mechanism, akin to theories of motor task-induced fatigue [138–140]. According to this view, perceived cognitive fatigue would act in anticipation of future functional alterations induced by prolonged task performance, to divert behavior away from the taxing activity [23]. It is assumed that several factors contribute to perceived cognitive fatigue. However, to the best of the authors’ knowledge, there has been no attempt so far to systematize these as was done for perceived motor fatigue with the three-dimensional dynamical system framework proposed by Venhorst et al. [5].

As mentioned above, one of the most relevant factors thought to contribute to perceived cognitive fatigue is the cognitive (or mental) effort invested in the task [9, 141]. It is thought that cognitive effort is associated with cognitive control, meaning that non-automated cognitive control-dependent processes, like the execution of difficult cognitive tasks, require cognitive effort [137]. As mentioned earlier, cognitive effort is perceived as costly as well as aversive and is only maintained or increased if it is expected to be beneficial. The costs of prolonged cognitive effort investment comprise the intrinsic costs related to cognitive control allocation per se as well as the opportunity costs that arise from forgoing other (more rewarding) behavior [137, 142]. However, there is also evidence that effort is not necessarily perceived as costly and can add value, meaning that the same outcome can be more rewarding when more and not less effort was invested [143]. It has been shown that several brain areas are activated when cognitive control and effort are exerted, which include the dorsal anterior cingulate cortex, anterior insula, lateral prefrontal cortex, and lateral parietal cortex [132, 144–146]. This is the case when a cognitive task has to be performed that requires sustained attention, maintenance of information in working memory, and/or the inhibition of prepotent responses. Similar to the perceived effort induced by motor tasks, cognitive effort perception and objective measures of effort investment during the same task can be modulated by homeostatic perturbations such as sleep deprivation [134] and heat stress [147], respectively.

It was argued that the costs of effort have a considerable impact on motivation, which drives the behavior of humans [9]. This interaction is intuitive, since motivation is not only directed towards a specific goal but also refers to the intensity (i.e., effort) with which this goal is pursued [143]. Müller and Apps [9] proposed that the psychophysiological processes associated with activity-induced state fatigue have an impact on motivation in two ways: they would cause direct changes in brain structures that motivate behaviors or would induce alterations in other systems, which are connected to or influenced by these brain areas.

Tasks that require sustained cognitive effort typically increase indices of sympathetic nervous system activity [148–150], which is interpreted to reflect an aversive affective response [143]. Indeed, it has been postulated that core affect, comprising affective valence (pleasure-displeasure) and arousal (activation-deactivation), changes during sustained cognitive tasks. This might occur at least in two ways: (1) increasing conflicts and errors during task execution result in negative affective valence leading to an increased effort to reduce conflicts and errors in order to achieve “cognitive comfort”. Alternatively, (2) repeated conflicts and errors induce negative affective valence signaling that the current task is unrewarding. The latter is associated with perceived cognitive fatigue, which is thought to direct the individual to other more rewarding activities or to reduce effortful conflict monitoring, especially during externally mandated cognitive tasks [151]. This view is in line with the notion that negative affect signalizes inadequate progress towards goal achievement [152], which was also discussed in the context of perceived cognitive fatigue [119]. Furthermore, it was proposed that the increasingly aversive sensation with time-on-task results from the effort-induced accumulation of opportunity costs that arise from forgoing other and more rewarding behavior [22].

Similar to motor tasks, performing sustained cognitive tasks requires self-regulation [153], which describes the dynamic process of bringing thinking and behavior in line with the desired goal [154]. During sustained cognitive tasks, individuals have to continuously self-regulate different aversive sensations (e.g., cognitive effort, frustration, boredom [155]), thoughts (e.g., related to task-termination or distractors), and behaviors (e.g., stopping the task or increasing the effort), with consequences for their cognitive performance. Self-regulation per se requires effort and relies on the integrity of executive functioning and in particular on the core executive functions, which can be classified into inhibitory control (i.e., response inhibition and interference control), working memory, and cognitive flexibility [66]. There are several ways in which people can self-regulate themselves to modify their sensations, feelings, thoughts, and behaviors in service of a personal goal including effortful self-control [143]. Although self-regulation and self-control are often used interchangeably [153], it was proposed that they refer to distinct processes [64]. While self-regulation refers to more general processes of goal-directed thoughts and behaviors, self-control can be defined as the process of overcoming predominant (pre-potent, automatic) response tendencies in favor of the desired goal [65]. Self-control is exerted during the execution of sustained cognitive tasks and requires motivation as well as attention [64]. This is in line with the view that performing a sustained cognitive task competes with motivational options (e.g., biological, emotional and/or alternative cognitive goals), which capture attention and have to be actively inhibited to maintain task performance [119].

In addition to these key-determinants, there are further important aspects that contribute to the psychophysiological state of an individual with potential consequences for perceived cognitive fatigue. For instance, it has been revealed that greater interest in a cognitive task resulted in less perceived cognitive fatigue despite a higher willingness to exert cognitive effort [156]. In the same manner, it was argued that the level of controllability modulates the aversive responses and perceived cognitive fatigue during sustained cognitive activities [119]. Furthermore, the execution of sustained cognitive tasks can be associated with changes in mood as well as with feelings like stress, anxiety, frustration, hopelessness, tension, and boredom [114, 141, 155, 157]. Some of these were shown to be related to cognitive task performance [155] and to modulate perceived cognitive fatigue [64, 119, 158]. For example, it was found that a task requiring the passive observation of strings of numbers resulted in higher boredom ratings, a steeper decline in affective valence, and higher perceived cognitive fatigue ratings compared to a cognitive task that consisted of adding three to each digit of a four-digit number [157]. Interestingly, passively watching strings of numbers was also rated as effortful, which was interpreted as the effort to keep paying attention. It was also proposed that boredom, caused by the low intrinsic attractiveness of the task itself, can also be responsible for a decrease in cognitive task performance [119], highlighting the interdependence between determinants of perceived cognitive fatigue and cognitive performance fatigue. Moreover, it was assumed that expectations based on previous experiences might influence the psychophysiological state and the psychophysiological adjustments during sustained cognitive tasks [159].

Alterations of an individual’s body homeostasis can also modulate perceived cognitive fatigue and its determinants (e.g., cognitive effort perception) during cognitive tasks. For instance, this was shown in response to heat stress [133, 147], sleep deprivation [134], and mouth rinsing with caffeine-maltodextrin [135]. These sources of influence also highlight the similarity between the constructs of sleepiness and fatigue. Confusion between those concepts is very common and it remains unclear to what extent the subjective assessment measures allow researchers to clearly tease them apart [160]. Besides these, there are further factors contributing to perceived cognitive fatigue that are increasingly studied and should be added to the list of potential determinants in the future.

### Modulating Factors of Cognitive Performance Fatigue and Perceived Cognitive Fatigue

Various modulating factors can influence the extent of cognitive performance fatigue and perceived cognitive fatigue (Fig. 1b). The main subject-specific factors include age, sex, the existence of diseases, and cognitive fitness. The extent of fatigue in the different domains is further determined by the characteristics of the cognitive task (e.g., type of task, duration, cognitive load), environmental conditions (e.g., temperature) as well as other homeostatic perturbations (e.g., thirst, sleep deprivation) [117, 133, 134, 143, 147, 161]. In the following sections, we will only discuss the most important subject- and cognitive task-specific factors that can modulate cognitive performance fatigue and perceived cognitive fatigue.

#### Subject-Specific Factors and Cognitive Performance Fatigue

Cognitive abilities, such as processing speed and executive functioning, decline with advancing age due to structural and functional changes within the brain (e.g., changes in white and gray matter volume, loss of synapses, dysfunction of neural networks) [162, 163]. It is thought that these age-related changes contribute to the increased (compensatory) brain activation observed during the execution of cognitive tasks [164], which was assumed to accelerate cognitive performance fatigue development [113]. However, the results of studies that have investigated the effect of age on cognitive performance fatigue are mixed and do not allow for a definite conclusion. For instance, it was found that reaction times remained constant in young and old adults after the execution of a working memory task performed for 60 min, while accuracy even increased in the elderly. The authors proposed that the potential decline in cognitive performance was countered by the learning effect [122]. This is in line with the results of Behrens et al. [11], who have found a progressive decline in reaction times with time-on-task, indicating a better task performance, during a 90-min inhibitory control task with no differences between young and old adults. In contrast, Terentjeviene et al. [113] found a similar progressively higher number of errors during an inhibition task performed over 120 min in younger and older males, which can be interpreted as cognitive performance fatigue. However, reaction times and intra-individual variability of reaction times only increased in young males indicating higher cognitive performance fatigue compared to the older males.

Since brain activation differs between sexes depending on the type of cognitive task [165, 166], it might be assumed that the extent of cognitive performance fatigue after a sustained cognitive activity is different between males and females. However, the results of experiments on that topic are inconclusive. For instance, there were no sex-differences in the error rate and reaction times, which remained constant over time, after performing a continuous performance test for 51 min [114]. This is consistent with the results of Wang et al. [166], who have investigated sex-differences in brain activation in response to psychological stress induced by an arithmetic task (i.e., serial subtraction of 13 from a four-digit number). The authors did not observe differences in the number of errors and completed subtractions between males and females. However, the task lasted only 12 min and was possibly not long enough to induce cognitive performance fatigue. In contrast to these studies, Noreika et al. [167] have shown sex-specific cognitive performance changes during a 90-min mental rotation task. They have revealed that accuracy increased, and response times decreased to a larger extent in males compared to females with time-on-task. However, since cognitive performance increased over time, these results did not indicate cognitive performance fatigue and might be biased by the learning or practice effect.

Since the extent of cognitive performance fatigue strongly depends on the structural and functional integrity of the central nervous system, it might be assumed that cognitive performance fatigue development is accelerated in patient populations, especially in those with neurological diseases affecting the central nervous system. However, studies investigating cognitive performance fatigue in different patient populations (e.g., people with multiple sclerosis, traumatic brain injury, depression, chronic fatigue syndrome) have often found an increase in cognitive task performance (e.g., decrease in reaction time, increase in accuracy) with time-on-task, indicating a learning or practice effect, with no or minor differences compared to healthy controls [168–171]. Despite similar increases in cognitive task performance, patients showed heightened cerebral activation in specific areas and an increased perceived cognitive fatigue [10, 170, 172] indicating a lower efficiency. In contrast, a decrease in cognitive performance measures was revealed during the execution of a four-block paced auditory serial addition test with an earlier drop in performance in people with multiple sclerosis compared to healthy controls [173]. Analogous results were obtained by other studies that investigated the effects of sustained cognitive tasks on cognitive performance fatigue in multiple sclerosis and healthy controls [174, 175]. A decrease in cognitive task performance was also found in stroke survivors while performing a 60-min inhibition task, which was, however, comparable to the cognitive task performance reduction of the healthy control group [176]. Moreover, Jordan et al. [177] have revealed that cognitive task performance declined during the execution of different sustained cognitive tasks in patients with myasthenia gravis but not in the healthy control subjects.

The discrepant findings presented above indicate that the effect of age, sex, and diseases on cognitive performance fatigue seems to be strongly influenced by the task characteristics (e.g., type of task, duration, cognitive load) as well as the parameters and tasks used for the assessment of cognitive performance fatigue (e.g., reaction times and accuracy during the fatiguing task or a separate task performed before and after the fatiguing task).

#### Characteristics of the Cognitive Task and Cognitive Performance Fatigue

The occurrence and evaluation of cognitive performance fatigue strongly depend on the type of task (e.g., working memory task, response inhibition task), further characteristics of the cognitive task (e.g., duration, cognitive load), and the task used to quantify cognitive performance fatigue [116–118, 178, 179]. For instance, Smith et al. [118] have examined the effects of three different cognitive tasks, each performed for 45 min, on measures of cognitive performance fatigue: (1) psychomotor vigilance task, (2) AX-continuous performance task, (3) Stroop task. While the first task required only vigilance, the other two tasks additionally relied on response inhibition. Cognitive performance (i.e., reaction times, errors, misses) was recorded during each task and additionally using a 3-min psychomotor vigilance task performed before and after each task. Interestingly, cognitive performance monitored during the respective tasks deteriorated only for the psychomotor vigilance task (i.e., increased reaction times and misses). In contrast, pre- and post-cognitive performance assessments with the 3-min psychomotor vigilance task revealed increased reaction times only after the AX-continuous performance task and the Stroop task. These data indicate that the detection and extent of cognitive performance fatigue strongly depend on the type of task as well as the task used to assess the potential change in performance.

Besides the effect of the type of cognitive task, other task characteristics (e.g., duration, cognitive load) can also influence the extent of cognitive performance fatigue. Although several studies have not found a decline in cognitive performance with time-on-task, it was demonstrated that cognitive task performance decreased with increasing task duration [21, 116, 117]. Furthermore, it was proposed that the cognitive load induced by the task determines the extent of cognitive performance fatigue [117, 180, 181]. Studies on this topic manipulated the cognitive load either by increasing the difficulty of the task (e.g., N-back paradigm: 0-back task [low cognitive load] vs 2-back task [high cognitive load] [180]) or reducing the processing time for the stimuli presented during the cognitive tasks [117]. However, the results of these studies are inconsistent. For instance, during a 30-min working memory task, Shigihara et al. [180] have observed an increase in reaction times with constant accuracy during the low cognitive load condition (0-back task), but not during the high cognitive load condition (2-back task). The authors have additionally tested the effect of the sustained cognitive tasks on the advanced trail making test performance and have found that the number of errors increased from pre to post for both conditions. These results again highlight the relevance of the cognitive tasks’ characteristics and the performance measures used to assess cognitive performance fatigue. Moreover, the results of Borragàn et al. [117] indicated that not the difficulty of the task (e.g., 0-back task vs 1-back task) but the processing time interval for the stimuli determines the extent of cognitive performance fatigue, with shorter intervals producing larger cognitive performance declines. However, irrespective of the task characteristics, there is a general critique that laboratory cognitive tasks are not ecologically valid and exhibit low intrinsic motivational value. Therefore, controllability of these tasks is low, people experience them as increasingly aversive, and might disengage from the tasks because they are not sufficiently important for them [22, 64, 119, 159].

#### Subject-Specific Factors and Perceived Cognitive Fatigue

Subject-specific factors like age, sex, and the presence of diseases might also modulate perceived cognitive fatigue and its potential determinants. For example, Wascher et al. [182] have observed a higher increase in perceived cognitive fatigue in young compared to old adults while performing a Simon task requiring inhibitory control for 21 min. Interestingly, young adults showed also a concomitant larger decrease in self-reported motivation. This is in line with the results of Terentjeviene et al. [113], who have found a higher rise of fatigue ratings during an inhibition task performed for 120 min in young compared to old adults. This was accompanied by a higher perceived cognitive effort and temporal demand in the young participants during task execution. The young adults additionally reported increases in tension and confusion as well as a decrease in vigor, which were not found for the older participants. These data collectively suggest that younger adults perceive sustained cognitive tasks as more effortful, demanding, and fatiguing than older adults, which is corroborated by the larger decreases in motivation and vigor as well as the increased tension and confusion. Since laboratory cognitive tasks are often performed using a computer and older adults have less experience with digital technology [183], these differences might be related to the higher intrinsic attractiveness of the task for older people. Nevertheless, it was also shown that older people with a low frequency of technology use have higher levels of computer anxiety [183]. Contrary to the findings of age-related differences, it has also been observed that the increase in perceived cognitive fatigue induced by a 90-min inhibitory control task was not different between young and old adults [11].

Studies on sex-differences in perceived cognitive fatigue also revealed partially inconsistent results. Some studies have not found differences in perceived cognitive fatigue between males and females after performing a 45-min Stroop task and a 51-min continuous performance test, which require response inhibition and sustained attention. Similarly, no sex-differences in the changes in cognitive effort, vigor, energy, tiredness, tension, calmness, and further self-reported data recorded during these tasks were reported [114, 184]. Nevertheless, others have observed higher increases in fatigue ratings during a 90-min mental rotation task in females compared to males, which depended on the menstrual cycle phase [167]. It was additionally revealed that perceived cognitive effort and perceived task difficulty were higher in females compared to males during both a high and low cognitive load condition involving arithmetic tasks [166].

There is evidence that perceived cognitive fatigue development in response to sustained cognitive tasks is higher in some diseases, especially those affecting the central nervous system [10, 171, 175, 185]. Higher increases in fatigue ratings during cognitive tasks have particularly been observed in people with multiple sclerosis [171, 175, 185]. It was further reported that the perceived workload (e.g., effort, mental, temporal, physical demand) induced by a 60-min stop-signal task was higher in stroke survivors compared to an age-matched control group [176]. In contrast, studies on other patient populations, such as people with chronic fatigue syndrome, depression, and myasthenia gravis, have not found differential changes in perceived cognitive fatigue ratings compared with healthy controls during sustained cognitive tasks [171, 177]. The discrepant findings on the effect of age and sex on perceived cognitive fatigue may partially be related to the tasks’ characteristics (e.g., type of task, duration, cognitive load), while task-induced perceived cognitive fatigue seems to be increased in some clinical populations such as multiple sclerosis.

#### Characteristics of the Cognitive Task and Perceived Cognitive Fatigue

It has been shown that perceived cognitive fatigue and its determinants can be influenced by the type of task (e.g., working memory task, response inhibition task) as well as other characteristics of the cognitive task (e.g., duration, cognitive load) [116, 117, 178]. The results of studies that have examined the effect of the type of cognitive task on perceived cognitive fatigue are inconclusive. For instance, O’Keefe et al. [178] have compared perceived cognitive fatigue after performing the AX continuous performance test for 90 min, which requires attention and response inhibition, and the TloadDback task executed for 16 min, which is a working memory dual task. The latter was applied with constant processing intervals and with shorter individualized processing intervals based on the maximal performance determined during an incremental TloadDback task. Although the task duration differed greatly, all cognitive tasks induced perceived cognitive fatigue, assessed with a visual analog scale, with the highest increase in the individualized TloadDback task condition. However, the AX continuous performance test induced a higher increase in perceived cognitive fatigue and a larger decrease in vigor assessed with the Brunel Mood Scale. These results were accompanied by higher sleepiness ratings and a larger drop in task motivation compared to the TloadDback task condition. Another study on this topic has compared perceived cognitive fatigue as well as its recovery in response to a psychomotor vigilance task, an AX-continuous performance task, and a Stroop task each performed for 45 min [118]. The tasks differed regarding their demands on vigilance and response inhibition. Although all tasks increased perceived cognitive fatigue, the authors concluded that tasks requiring response inhibition appeared to induce perceived cognitive fatigue for a longer duration than a simple vigilance task.

With regard to other task characteristics, it was often found that perceived cognitive fatigue progressively increases with time-on-task [116, 185]. Moreover, it was shown that altering the cognitive load by the difficulty of the task (i.e., N-back paradigm: 0-back task [low cognitive load] vs 2-back task [high cognitive load]) resulted in comparable increases in the fatigue ratings after both tasks. In line with this, Borragàn et al. [117] found a similar increase in perceived cognitive fatigue in the low and high cognitive load condition, when the cognitive load was manipulated by the number of items to be processed during the task. However, when cognitive load was modulated by decreasing the processing interval for the stimuli, the high cognitive load condition induced a higher increase in perceived cognitive fatigue compared to the low cognitive load condition. The authors concluded that the processing time interval during cognitive tasks is more relevant for perceived cognitive fatigue development than the number of processed items. In contrast to this, it was also shown that perceived cognitive fatigue cannot only result from performing a sustained cognitive task (i.e., adding three to each digit of a four-digit number for 20 min) but also from passively observing strings of numbers intended to induce boredom [157]. More specifically, perceived cognitive fatigue was even higher in the boredom condition compared with the cognitive task condition despite lower cognitive effort ratings. This might be related to the steeper decline in affective valence ratings and the lower task interest ratings in the boredom condition. Indeed, higher interest in a task has been shown to induce less perceived cognitive fatigue [156]. These data highlight the importance of the characteristics of the cognitive task for the development of perceived cognitive fatigue and its determinants. Moreover, the individuals’ attitude towards the cognitive task (e.g., interest) seems to modulate the perceptual, affective, and cognitive responses.

## Unraveling the Interactions Between Performance Fatigue and Perceived Fatigue: Recommendations for Future Research

The updated framework covers the different dimensions of task-induced state fatigue and the involved mechanisms (Fig. 1). Thereby, the interdependence of performance fatigue and perceived fatigue as well as their determinants is acknowledged and highlighted. There is no single factor primarily determining performance fatigue and perceived fatigue in response to motor and cognitive tasks. Instead, the relative weight of each determinant and their interaction depends on several modulating factors (e.g., age, sex, diseases, fitness, characteristics of the motor and cognitive task).

### Unraveling the Interactions Between Motor Performance Fatigue and Perceived Motor Fatigue

Although the mechanisms of motor performance fatigue are not yet fully elucidated, there are extensive data on the changes in the nervous system and muscle during motor tasks contributing to the decline in motor performance [16, 17, 25, 26]. In contrast, the mechanisms underlying perceived motor fatigue and their interactions with motor performance fatigue received less attention. Therefore, future research should not only investigate the neural and muscular mechanisms driving motor performance fatigue but also aspects of perceived motor fatigue and the corresponding (neuro)physiological correlates in detail. The combined measurement of changes in maximal and/or submaximal motor performance, their neural and muscular determinants as well as subjective perceptual, affective, and cognitive responses will help to understand state fatigue in different populations and in response to different motor tasks. This approach can assist in investigating the motor task-induced perceptual differences between individuals and exercise protocols (e.g., some experience exercise-induced muscle pain, whereas others primarily perceive breathing discomfort during the same motor task) and their effects on affective and cognitive processes as well as motor performance fatigue. This is of special importance for clinical populations suffering from an increased prevalence of motor performance fatigue and perceived motor fatigue (e.g., multiple sclerosis, chronic obstructive pulmonary disease, rheumatoid arthritis) [2, 6–8, 12].

There are a few studies available that have assessed neural and muscular contributions to motor performance fatigue in parallel with ratings of perceived motor fatigue, effort perception, and affective valence to study their interactions [42, 186]. For instance, Greenhouse-Tucknott et al. [42] investigated the neuromuscular as well as perceptual and affective mechanisms responsible for the reduced endurance performance of the knee extensors following prior upper body motor activity. They have shown that prior submaximal hand grip exercise reduced the time to exhaustion during a submaximal isometric contraction of the knee extensors without altering neuromuscular function. However, they have found increased perceived motor fatigue as well as effort perception ratings and a reduced affective valence in the ‘prior exercise condition’ compared to a passive control condition. Thereby, effort perception and affective valence were correlated with time to exhaustion and the ratings of perceived motor fatigue. These findings indicate that prior handgrip exercise limited single-joint endurance performance of the knee extensors primarily by the interactions between perceived motor fatigue, effort perception, as well as affective valence and not by a decreased neuromuscular function. Similar approaches should be adopted in the future to investigate the interactions between motor performance fatigue and perceived motor fatigue in different populations, particularly in those suffering from diseases.

Besides the combined investigation of motor performance fatigue, perceived motor fatigue, and the underlying mechanisms, the determining factors can be manipulated to elucidate their causal involvement in the development of state fatigue in different populations and in response to various motor tasks. For that purpose, different interventions can be used aiming to modify the physiological and psychological regulatory processes during fatiguing motor exercise. For instance, neuromodulation techniques like tDCS are suitable to alter cortical excitability and to investigate the effects of changed neural properties on motor performance fatigue and perceived motor fatigue [67]. Furthermore, other interventions can be applied to modify neural as well as muscular properties (e.g., triggering ‘mental fatigue’ by a sustained cognitive task, supplements like caffeine or dietary nitrate, ischemic preconditioning) to investigate their effects on the different dimensions of motor task-induced state fatigue [18, 51, 52, 109]. Interventions aiming to modulate the psychological determinants of endurance performance have also been shown to induce changes in motor performance and the perceptual responses to fatiguing exercise [48]. These strategies could be used to investigate the role of cognitive processes in the interpretation of perceptual responses and the change in affective valence emerging during fatiguing motor exercise.

Motor performance fatigue can be assessed using maximal and submaximal motor performance measures. Maximal performance tasks (e.g., maximal voluntary contractions jumps) are suitable to monitor changes in the maximal capacity of the neuromuscular system to produce force or power in response to a fatiguing motor task [187, 188]. In addition, the variation of submaximal motor performance is also an indication of motor performance fatigue (e.g., force fluctuations during submaximal isometric contractions, coefficient of variation of kinematic gait parameters) [11, 189, 190].

The neural and muscular mechanisms contributing to motor performance fatigue can be investigated with different non-invasive techniques. Neural adjustments (i.e., muscle activation) can be quantified, for example, with transcranial magnetic stimulation, peripheral nerve stimulation, electromyography, functional near-infrared spectroscopy, and electroencephalography [187, 191, 192]. Moreover, functional magnetic resonance imaging is suitable to monitor changes within cortical and subcortical structures during motor exercise [193]. The contractile function of muscles can be validly quantified using peripheral nerve stimulation [187], while changes in muscle oxygenation and muscle metabolism can be measured with near-infrared spectroscopy and 31-phosphorus magnetic resonance spectroscopy, respectively [194, 195]

In addition to these measures, perceived motor fatigue as well as the contributing factors should be comprehensively assessed before, during, and after fatiguing exercise. For this purpose, different scales and questionnaires can be used according to the focus of the respective study. Perceived motor fatigue can be assessed with the ratings of fatigue scale [13], while effort perception and exercise-induced pain/discomfort perception can be quantified with 15-point Borg scales and/or category ratio scales (CR10 and CR100). These measures should be applied together with standardized wording as described elsewhere [5, 38]. Furthermore, the attentional focus during fatiguing motor exercise should be recorded as an index for the motor task intensity-dependent attentional shift from an external focus on the surrounding to an internal focus on the bodily sensations [196]. These aspects should be quantified in conjunction with affective valence and arousal, recorded with the feeling scale and felt arousal scale, respectively, as indicators of the motor task-dependent homeostatic perturbations [40, 60]. It has been shown that these aspects can influence perceived motor fatigue and performance during fatiguing motor tasks. Moreover, they reflect the motor task-induced homeostatic perturbations in various physiological subsystems and are thus indicators of the physical demands.

Besides these core measures of perceived motor fatigue, additional scales and tests should be used according to the aim of the respective study. This should be done to investigate the role of self-regulation capacity, executive functioning [41, 197, 198], and other determinants for motor performance fatigue as well as perceived motor fatigue.

### Unraveling the Interactions Between Cognitive Performance Fatigue and Perceived Cognitive Fatigue

The interactions between cognitive performance fatigue and perceived cognitive fatigue have been investigated more comprehensively and in greater detail compared to those between motor performance fatigue and perceived motor fatigue. Accordingly, many studies in this field have recorded both changes in cognitive performance as well as in the perception of fatigue during and after sustained cognitive tasks. However, it must be pointed out that in studies which have measured cognitive performance on the same task as the one used to induce fatigue, evidence for a decline in cognitive task performance was frequently missing [119, 199]. The lack of a systematic decline in cognitive performance with time-on-task was often attributed to an increased compensatory cognitive effort or to a learning effect that would lead to a performance increase overcoming the performance decline induced by fatigue [119–122]. In contrast, increases in perceived cognitive fatigue with time-on-task have been shown very consistently across many different conditions (e.g., types and loads of cognitive tasks) [117, 118, 178, 180]. Therefore, future studies should use separate cognitive tasks to induce and measure cognitive performance fatigue as already done by some studies [23, 24, 118, 126, 180]. This approach might bypass the influence of a decreased motivation or learning effect and has typically led to more consistent correlations between the cognitive performance decline and perceived cognitive fatigue [24, 126].

Furthermore, the effect of distinct types (e.g., inhibitory control, working memory, or cognitive flexibility task) and loads of cognitive tasks on cognitive performance fatigue and perceived cognitive fatigue should be investigated in more detail. There is evidence that different cognitive tasks induce specific declines in performance measures depending on the assessment task. For instance, Smith et al. [118] have shown that performance decreased with time-on-task only for the psychomotor vigilance task, but not for the AX-continuous performance task and Stroop task, each performed for 45 min. On the contrary, pre and post cognitive performance assessments with a 3-min psychomotor vigilance task revealed only increased reaction times after the AX-continuous performance task and the Stroop task. However, perceived cognitive fatigue increased in all conditions, even though it tended to persist longer after the tasks requiring more response inhibition. Moreover, cognitive performance fatigue and perceived cognitive fatigue measures were also shown to be sensitive to the manipulation of the cognitive load [117, 200]. Consequently, future studies on this topic should not only examine the effects of diverse types of tasks on cognitive performance fatigue, perceived cognitive fatigue, and their neural correlates but also the influence of varying cognitive loads.

Furthermore, it is likely that the level of overlap between the fatiguing and the assessment tasks, in terms of the cognitive processes involved, is also crucial [201]. Therefore, it appears essential that future studies assess performance before and after the fatiguing cognitive task not only with cognitive tasks requiring similar cognitive processes, but also with tasks that involve different processes. These experiments should further take the impact of the cognitive load, the nature of the cognitive processes involved, and the degree of process overlap with the fatiguing task into account. As stated above, other important sources of influence are mood and emotional variables, like stress, anxiety, frustration, hopelessness, tension, and boredom [114, 141, 155, 157], which were shown to modulate perceived cognitive fatigue [119, 157, 158] and cognitive task performance [155]. Therefore, it seems mandatory to quantify these aspects and to analyze their effects on cognitive performance fatigue, perceived cognitive fatigue, and their neural correlates.

Due to the discrepant findings, the influence of subject-specific factors (i.e., age, sex, the presence of diseases) on cognitive performance fatigue and perceived cognitive fatigue also require further investigation. Nevertheless, it seems that cognitive task-induced perceived cognitive fatigue is higher in some patient populations [171, 175, 185].

To address the causal relationships, neurophysiological and psychophysiological determinants of cognitive performance fatigue as well as perceived cognitive fatigue can also be modulated experimentally. For instance, it was shown that anodal tDCS applied to the right parietal cortex counteracted the cognitive performance decline during a 40-min visual vigilance task in healthy controls and people with multiple sclerosis but had no effect on perceived cognitive fatigue [202]. Similar effects were observed in multiple sclerosis patients after stimulating the left dorsolateral prefrontal cortex with anodal tDCS [203]. In addition, investigating the effects of different neuromodulatory substances such as caffeine on cognitive performance fatigue, perceived cognitive fatigue [135], and its neurophysiological correlates can help to unravel their interdependence.

As outlined above, the detection and quantification of cognitive performance fatigue strongly depend on the assessment task and the considered variables (e.g., reaction times, accuracy, variability) [112, 118]. Perceived cognitive fatigue as well as other task-induced sensations and emotions can be captured, for instance, using visual analog scales and/or standardized questionnaires on mood (e.g., Brunel Mood Scale), workload (e.g., NASA Task Load Index), and activation states (e.g., Activation Deactivation Adjective Check List) [10, 11, 112, 114, 118, 155, 157]. These measures should be combined with techniques suitable to record brain activity (e.g., functional magnetic resonance imaging, electroencephalography, functional near-infrared spectroscopy) [113, 125, 128] and autonomic nervous system function (e.g., heart rate variability, pupil diameter) [11, 204, 205] to learn more about the interactions of cognitive performance fatigue, perceived cognitive fatigue, and their neural correlates.

### Unraveling the Interactions Between Motor Performance Fatigue, Perceived Motor Fatigue, Cognitive Performance Fatigue, and Perceived Cognitive Fatigue

Ample evidence points to the interactions between the different dimensions of activity-induced state fatigue. For instance, Marcora et al. [18] have shown that performing a 90-min response inhibition task decreased time to exhaustion in a subsequent constant-load cycling task at 80% peak power output without differences in the physiological variables compared to a control condition (watching a documentary). However, effort perception during exercise was higher after performing the sustained cognitive task leading the authors to the conclusion that the participants reached their maximal tolerable effort level earlier and subsequently disengaged from exercise. Moreover, it was observed that 60-min constant-load cycling at 90% ventilatory threshold resulted in higher reaction times during a 40-min visual working-memory vigilance test compared to the group that did not exercise before [206]. These data indicate that fatiguing cognitive or motor activities seem to modulate the performance and perceptions during a subsequent fatiguing motor or cognitive task, respectively. Evidence for the interactions between the different dimensions of activity-induced state fatigue also arises from experiments that have investigated the psychophysiological adjustments and performance changes in response to sustained motor-cognitive dual tasks [100, 186, 207]. These studies have found a decreased time to exhaustion during submaximal motor tasks when a concurrent cognitive task had to be executed (e.g., arithmetic task, N-back task). Additionally, time to exhaustion during a fatiguing motor-cognitive dual task tended to be shorter for a high compared to a low cognitive load condition. This was accompanied by a higher reduction in muscle activation of the knee extensors (i.e., voluntary activation assessed with peripheral nerve stimulation) as well as an increased heart rate and pupil diameter in the dual-task conditions compared to the single motor task condition. As expected, cognitive effort perception scaled with the level of cognitive load, but, surprisingly, effort perception associated with the motor task was greater in the high cognitive load condition compared to that recorded during the single motor task condition [186]. These data have impressively shown that the different domains of activity-induced state fatigue interact with each other. Since there is an overlap of brain structures involved during the execution of fatiguing motor and cognitive tasks (e.g., prefrontal cortex, anterior cingulate cortex) [9, 208], it was argued that these represent the mechanistic basis for the observed effects [186, 209]. Thereby, the degree of overlap between cognitive processes required for the respective motor and cognitive task might mediate the detrimental effects on performance and perceptions [9, 159, 209]. Consequently, future studies should investigate the effects of diverse types (e.g., inhibitory control, working memory, or cognitive flexibility task) and loads of cognitive tasks performed prior to or during motor exercise on performance fatigue and perceived fatigue measures. These effects should also be studied in relation to various motor tasks (e.g., whole-body and single-joint exercise in different intensity domains). Conversely, the impact of different fatiguing motor tasks performed prior to various fatiguing cognitive tasks relying on distinct cognitive processes should be examined.

The mechanistic basis for the interactions between motor performance fatigue, perceived motor fatigue, cognitive performance fatigue, and perceived cognitive fatigue can be probed with the methods mentioned in the respective paragraphs above. However, the quantification of some perceptual responses to motor and cognitive tasks requires specific scales and a clear description with standardized wording. This is of particular importance for the measurement of perceived fatigue and effort in response to motor, cognitive, or motor-cognitive dual tasks. Although it was proposed that the feeling of fatigue arising from exertion might be similarly induced by motor and cognitive tasks [9], results of studies indicated that perceived motor fatigue and perceived cognitive fatigue represent different perceptual domains [210, 211]. The same applies to effort perception, which can be related to either to the motor or the cognitive task [186].

## Conclusion

Performance fatigue and perceived fatigue as well as their determinants are interdependent and should not be considered in isolation. Consequently, there is no single factor primarily determining performance fatigue and perceived fatigue in response to motor and cognitive tasks. Instead, the relative weight of each determinant and their interaction depend on body homeostasis (e.g., wakefulness, core temperature) and several modulating factors (e.g., age, sex, diseases, characteristics of the task). Therefore, a combined assessment of performance fatigue and perceived fatigue measures as well as its (neuro)physiological correlates is required to unravel the psychophysiology of motor and cognitive task-induced state fatigue. This will help to better understand the interactions between the different dimensions of fatigue and their impact on human performance, which is necessary to design effective interventions for increasing exercise tolerance and human performance in healthy and clinical populations.
